# Decoding climate anomalies—From scientifically sound to societally relevant

**DOI:** 10.1073/pnas.2321154122

**Published:** 2026-10-26

**Authors:** Molly E. Brown, Elena Tarnavsky, Markus Enenkel

**Affiliations:** ^a^ Department of Geographical Sciences, University of Maryland, College Park, MD 20742; ^b^European Commission, Joint Research Centre (JRC), 21027 Ispra, Italy; ^c^ Global Water Practice, The World Bank, Washington, DC 20433

**Keywords:** climate data, heatwave, drought, decision-making

## Abstract

As climate impacts worsen, decision-makers, researchers, and public sector institutions increasingly need reliable, relevant, and appropriate information about emerging weather and climate anomalies. However, with the ever-expanding choice of datasets based on ground observations, satellite data, and models, it is critical to not only use the best dataset for a specific decision-making need but to avoid misinforming and even derailing decision-making due to dataset selection. The main objective of this perspective is to illustrate the importance of understanding datasets used to translate climate data into actionable knowledge, supporting the next generation of science- and data-driven climate risk management instruments, projects, and global initiatives. Through the lens of three use cases, we illustrate what data quality means for different applications, the implications of choosing, aggregating, and combining different climate datasets, and the potential consequences of dataset and baseline choice on decision-making. Choosing different datasets often leads to trade-offs that decision-makers need to be aware of, for instance, between the spatial resolution of a dataset and length of the data record. Here, we discuss the implications of selecting the appropriate time step, resolution, and dataset and provide examples of how these choices affect the identification of societally relevant anomalies.

The promise of data on climate, derived from ground observations, Earth-observing satellites, or models, is clear: insights into the local impact of continental or global climate processes via quality-controlled, harmonized time series or maps (1). Due to the advent of scalable cloud processing and storage infrastructure (2), user-friendly, online interfaces (3) facilitate understanding of historical climate conditions and allow current anomalies to be put into a historical context (4). In many cases, petabytes of data, decades of climate records, satellite data assimilated into climate models, advanced calibration, and validation are needed to inform decisions with high socioeconomic importance. These decisions could involve actions such as triggering evacuations due to a risk of flooding (5), response planning in anticipation of severe storms (6), distribution of drought-resistant seeds in a humanitarian domain (7, 8), and/or issuing drought early warnings or index-based insurance products based on modeling tools that underpin international humanitarian decision-making (9). From a risk financing perspective, reaching critical thresholds in a data-driven parametric insurance product usually corresponds to the release of preagreed funding to farmers or governments (10). With regard to climate data for decision support, each stakeholder community has specific needs which vary depending on the balance between livelihood, natural resources, economic orientation, and exposure to climate extremes.

The challenge for these applications is that the people developing decision-making systems and managing socioeconomic risk are mostly not the ones developing climate data and early warning systems (11). This disconnect leads to misconceptions or misunderstandings regarding the suitability, characteristics, strengths, and limitations of climate observations. We focus on historical climate observations over the past four decades (12), not on modeled climate predictions or projections. To illustrate these challenges, we present three case studies demonstrating the consequences of the data quality, choice of climate indicator, and the impact of differing climate baselines. Engaging with experts such as those available in every country in the local National Weather and Hydrological Services (NWHS) is critical to ensuring context and decision-making is central to climate analysis. NWHS are increasingly focused on generating more actionable products, engaging meaningfully with diverse end-users, and moving toward impact-based forecasts (13). Focusing on the impact of data choices is crucial for climate preparedness, as data are often used as triggers for specific response and adaptation actions.

Climate impacts are already extensive, multisectoral, and context specific (14). Understanding how to identify weather anomalies that are likely to result in negative impacts on individuals, households, or communities is very challenging. Direct and indirect health consequences of temperature and precipitation extremes (12) have been documented across all climate zones, with impacts being particularly important in low-income countries (15), for pregnant people (16), or disadvantaged populations in wealthy countries such as the United States (17, 18). Heatwaves, such as prolonged periods over 30 degrees Celsius may cause excess mortality, morbidity, and child development, particularly in tropical regions or in urban areas (19, 20). Increases in both extreme precipitation and total precipitation have resulted in increases in deaths and damage from flooding (21), destruction of communities via landslides (22), and widespread acknowledgment of the role of land use in urban areas on increased surface water flows (23).

However, determining who is at risk or how much to invest in response to extremes requires spatial and temporal granularity of the weather data, as well as a clear decision framework which connects hazards to decisions that have financial consequences. For example, in the European Union, the U-Adapt! Framework has five different domains: urban heat island effect, heat event exposure, individual or community sensitivity to heat, coping capacity, and heat event adaptive capacity. These goals are, in turn, folded into twenty-two adaptation objectives, each of which has associated costs. Few governments have either the resources or the institutional capacity to implement such a complex system (24). Determining who may be at risk from a climate event and how extreme it may be requires careful analysis of anomalies. The next two sections focus on how to detect anomalies and transform them into societal impacts.

## Complexities of Detecting Anomalies

Describing impacts of climate hazards typically starts with detecting anomalies. Data that describe climate impacts are typically focused on determining anomalies and/or trends (25, 26) and seek to quantify a hazard being experienced by a community, region, or sector. In the context of the international humanitarian sector, for example, drought assessment tools are used such as the Water Requirements Satisfaction Index (WRSI) model to translate climate into crop production impacts through the use of anomalies of drought-relevant indicators (27, 28) and to inform index-based drought insurance (9, 10). However, determining which data to use to capture these anomalies is of utmost importance to government agencies, policymakers, disaster risk managers, and crisis response units. Potential issues that occur with dataset selection include the following:

- Using too short or too long of a baseline as a reference for “normal” conditions to determine the severity and spatial extent of an anomalous event can circumvent efforts to understand and detrend the data or bias decision-making (29);
- An overwhelming focus on high spatial resolution vs. data quality when providing drought hazard maps (30) because, independent from their quality, higher-resolution climate data seem easier to understand and interpret for layman users;
- Assumptions about direct, linear spatial, or temporal relationships between climate shocks (e.g., drought) and socioeconomic conditions (e.g., food insecurity) (31); or
- Sensitivity to different rainfall datasets or reference crops of tools such as the WRSI model that are used to translate climate variables into drought indices and/or crop production impacts and inform parametric drought insurance design (9).

Even if climate data fit the type and scale of the application, which is often not the case (32), the issue of translating hazard data into socioeconomic impacts remains—particularly in low-income, data-scarce countries where data on local vulnerabilities and disaster impacts are missing. The global Emergency Database (EM-DAT), for instance, has large gaps in its data representing the human and economic impact of disasters (33). Drawing on a case study on flood forecasts in Uganda, Mitheu et al. (5) provide a set of recommendations for using impact databases such as EM-DAT and the key factors to be considered, i.e., inclusion criteria, consistency, representativeness, and underreporting in public sources due to accessibility issues, among others. Instead of socioeconomic data specific to a region or time of year, annual national-level UN statistics are frequently used, which can greatly underestimate the impact of the event on affected communities. Alternatively, Demographic and Health Survey (DHS), the World Bank’s Living Standards Measurement Study (LSMS) or UNICEF Multiple Indicator Cluster Surveys (MICS) provide individual-level information which can be used to determine the impact of a hazard. However, none of these information sources are harmonized with each other in terms of methodology and are not synchronized regarding the timing of data collection or choice of collected variables (34), creating an additional challenge to link climate shocks to socioeconomic impacts.

There are many positive examples of academia and practitioners collaborating based on a joint vision (35–37). However, climate science translators (38), (30) have a lot of work to do to foster communication (39) and collaboration between climate science, humanitarian decision-support, hazard policy, and decision-making. The success of these efforts to a large extent relies on the capacity to translate robust climate anomalies into actionable, societally relevant knowledge.

## Complexities of Defining Societally Relevant Anomalies

Identifying critical thresholds in climate anomalies (i.e., triggering levels) is vital for determining societally relevant anomalies. Although climate anomalies are defined as the departure of a climate parameter such as temperature or precipitation from its long-term average for the area concerned, understanding whether a particular climate anomaly is societally relevant is much more complex. For example, Smit et al. (40) used three questions to frame the impact of climate anomalies: i) How anomalous was the event?, ii) who or what is affected by the event? and iii) how does the impact of the event occur? The impact depends fundamentally on the characteristics of the system of interest, including who or what is affected. For example, the 2003 France heat wave involved temperatures that exceeded 40 degrees C for over eight days, which if it had occurred in Phoenix Arizona, would have been below average for July. However, in France, mortality was very high due to a lack of coping capacities at a large scale, such as the lack of air conditioning, combined with systemic weaknesses, such as the type of residential buildings in most of the country, the social isolation of many affected, and an aging population.

Translating climate information into impacts that are relevant for a specific location and context is an important consideration when determining data quality, climate indicator choice, and baseline time period for calculating anomalies. Independently from the chosen dataset, indicator, and baseline, “extreme” events are rare by definition. They represent the statistical tail of a climate dataset’s value distribution over time. Analyzing the tails, for instance, to understand return periods (i.e., period between two events with a comparable severity) or via more complex analyses, helps in identifying and characterizing these rare events, their socioeconomic impacts, and preparing for them. Decision-makers need to understand what the values of the chosen climate variables mean in the context of the target region and the specific decision to be made.

The arid regions of Somalia, for instance, receive a monthly average rainfall of 20 to 25 mm at the maximum (May). A few hundred kilometers away, in the Ethiopian highlands of Amhara, farmers are used to more than 300 mm in August. There are many drought indicators, which translate raw data into a metric that puts the value for the chosen period into a historical perspective. This historical perspective, represented by all values from the past 30 years, ending at the most recent period, represents climatology. Longer climatologies are generally considered more robust because they are less susceptible to short-term variability. Using a 5-y rainfall climatology, which includes three very dry years, as the baseline for the upcoming season in Somalia means that conditions will only appear “extreme” if they are worse than the low average of the short climatology (41). Using a longer climatology helps to better distribute these three extreme years over the entire value range of the previous 30 y at the cost of introducing long-term climate trends. These trends could be related to climate change but may also be due to changing meteorological station data, technical issues regarding the retrieval process, or other factors leading to complex feedback loops between the land surface and the climate (e.g., deforestation).

Thus, understanding the consequences of the data quality, choice of climate indicator, and severity and extent of impact is crucial for climate change adaptation and forward-looking humanitarian instruments (e.g., anticipatory action), as data are increasingly used as triggers for specific preparedness and response actions. Here, we provide three case studies that seek to illustrate the challenges of selecting datasets that can quantify socioeconomic climate impacts.

## Case Study #1: Data Quality in Northern Senegal

**Fig. 1.**
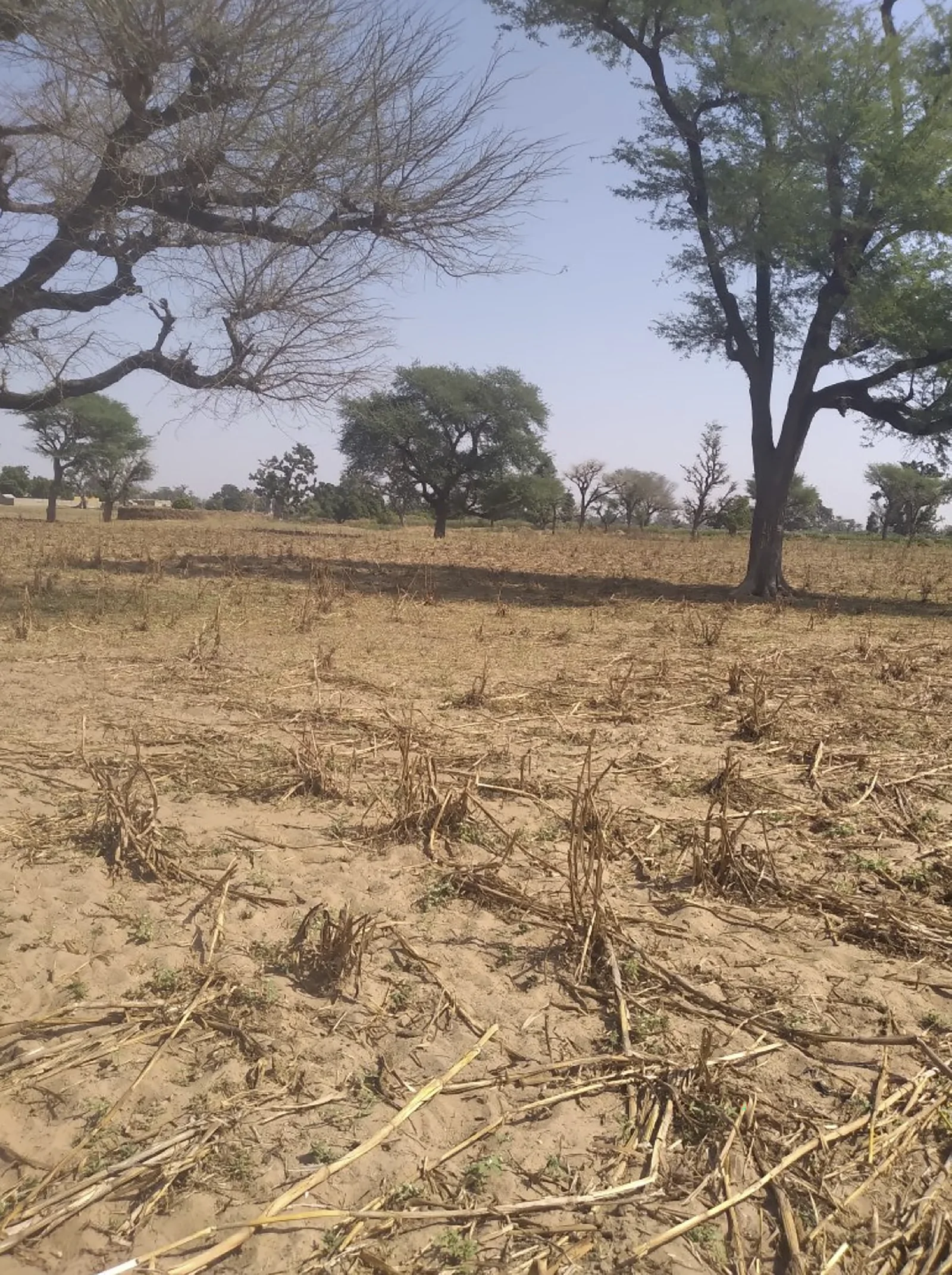
What is a field? Senegal millet field with acacia trees. Photo by the author.

Climate change is already causing significant impacts around the world (14). Hazards are being felt in many areas with less-favorable climates, soils, and infrastructure for agricultural production, such as in northern Senegal. Marginal agricultural lands are often located in regions with high median temperatures, low and highly variable rainfall, and with little to no access to agricultural technology (42). Identifying climate data that can provide appropriate information to determine changes in productivity in these systems requires a careful understanding of the agroecosystem. For example, in northern Senegal, agricultural production occurs on very small fields that are cultivated across a highly dynamic, complex, heterogeneous agricultural landscape (Fig. 1). Current earth observing systems are either too coarse or not frequent enough to capture production on these very dry, tiny, sparsely cultivated fields (43, 44). Poorly attuned data will result in a failure to capture actual losses by farmers, making it very challenging to design social safety nets and agricultural insurance programs that meet the needs of these communities. In arid regions like northern Senegal, agriculture is very sensitive to small increases in rainfall, moisture, or evapotranspiration. A single moderate rainfall event often looks like a huge anomaly in climate data using standard thresholds. Since agriculture insurance programs often use meteorological or remotely sensed monitoring systems to trigger payouts, a single rainfall event can prevent a payout even if the moisture cannot be effectively used by crops.

**Fig. 2.**
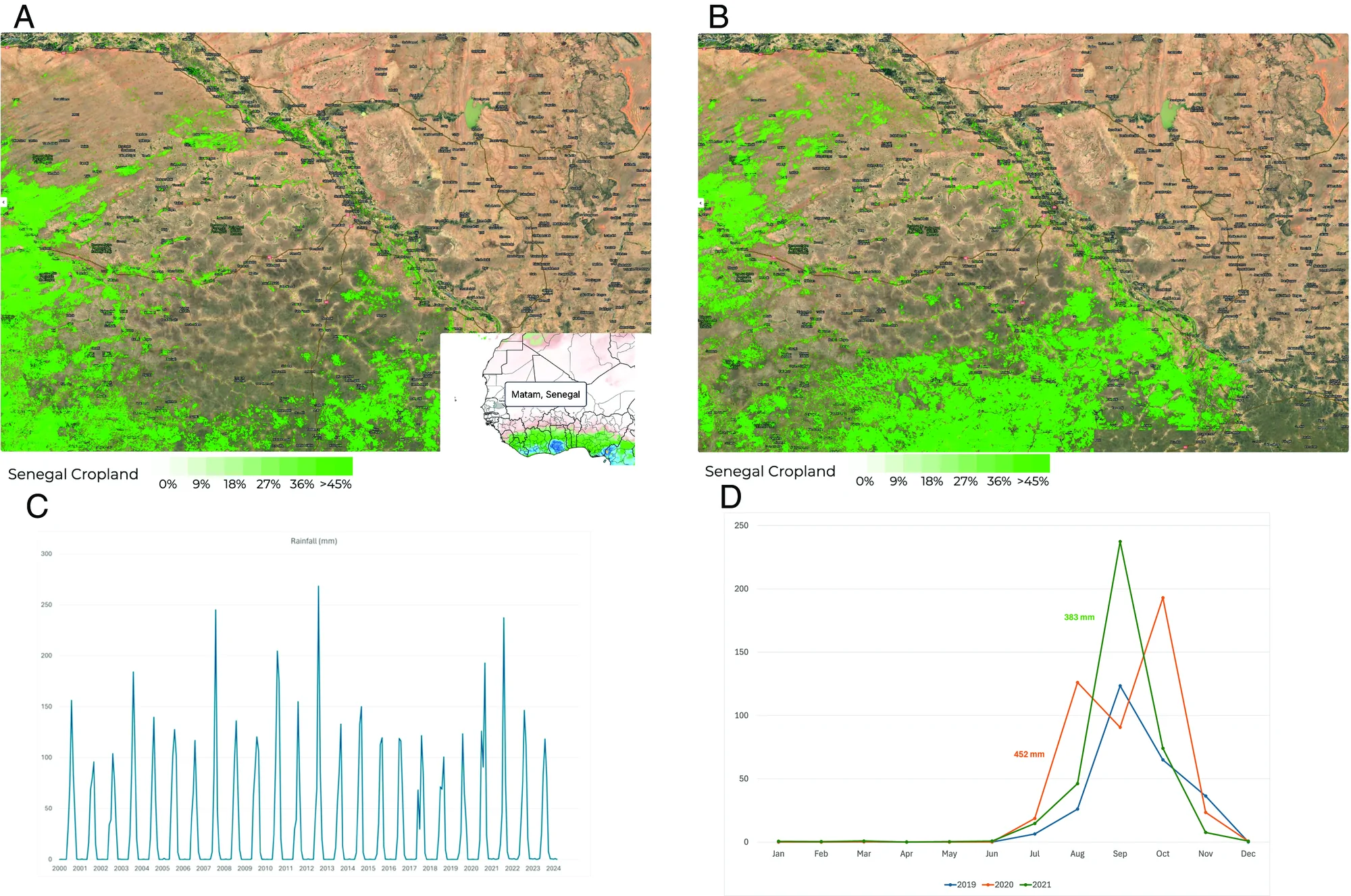
Data from northern Senegal Matam district, an area with primarily agricultural communities who rely on extremely marginal rainfall. (*A*) Cropped area maps from 2019 derived from Sentinel 2a/b, (*B*) Cropped area map from 2021 derived from Sentinel 2a/b, (*C*) Rainfall time series from 2000–2024 from CHIRPS data (47), (*D*) Rainfall monthly data and totals for 2019–2021 from CHIRPS data.

Although the quality and abundance of remotely sensed rainfall, temperature, and vegetation data have never been higher (45), it is still not sufficiently sensitive to capture variations in productivity in arid agricultural landscapes such as those in northern Senegal (Fig. 2). Only commercial datasets offer higher temporal frequency, but using those data requires purchasing access to the data as well as investing in significant up-front analytical expenses to set up a system that can clean, remove cloud and aerosol contamination, geometrically correct, spectrally calibrate, and analyze the data. For example, the 3 m spatial resolution PlanetScope data can have considerable image-to-image shifts between different images and in comparison to locations on the ground that must be corrected before the data can be used within insurance programs (46).

Even with higher-resolution optical remote sensing data, there is a challenge to connect reflectance recorded at the sensor to agricultural productivity in smallholder systems (48). In a study done in Eastern Uganda, Lobell et al. (49) showed very little correspondence between satellite-based approaches to estimating crop yields, data from crop cuts, and farmer-reported yields. They found a much higher relationship between full-plot crop cuts and satellite-based approaches due to much higher sample sizes. In very arid ecosystems such as northern Senegal, farmer-reported yields are even less reliable and are often extremely uncertain given the high interannual variability of rainfall and irrigation water availability.

Carcedo et al. (50) highlight, in particular, the weakness of crop model specifications relevant to arid, monsoonal ecosystems such as the northern areas of Senegal. Without high-resolution, agriculturally relevant data, drought risk financing programs cannot establish credible baselines at field scale to drive accelerated assistance in the face of floods, droughts, or tropical storms (51, 52). Climate data underpin a drought risk financing framework that allows the semiautomated design of triggers based on preprocessed data that are cross-checked/validated by reported drought years. We can use the convergence of evidence of different satellite-derived datasets, for example, soil moisture as a stand-alone variable or shadow model [strengthening the parameters driving the Water Requirement Satisfaction Index (27)], modeled estimates of soil moisture to predict near-future vegetation health, statistical analysis of various satellite-variables and annual crop yield data from FAO (53), and/or vegetation health. However, in regions with very little rainfall, even small rainfall events can result in significant vegetation anomalies that can trigger a payout even if the crop does not benefit.

Current rainfall, temperature, and cropped area data are just not good enough to determine the impact of drought on livelihoods in arid landscapes at the household level. For example, Kerner et al. (54) found that nearly all the land cover maps available for the West African Sahel were unable to capture observed cultivated areas, with most land cover maps predicting no cultivation despite widespread agricultural livelihoods, such as those present in northern Senegal (54). Given that the minimum rainfall totals that the most widely cultivated small grain in the region (pearl millet) can survive is 400 mm per year, the northern Senegal region has marginal rainfall even in a good year (55). The interannual variability of rainfall is significant, with almost 200 mm more rainfall in Matam region in 2020 than in 2019, with the rainfall in all three years just at the minimum level that millet can be cultivated (Fig. 2 *C* and *D*). Although the cropped area maps shown in Fig. 2 *A* and *B* show increased cropland in the Matam region of Senegal from 2019 to 2021, this could be simply pasture or natural vegetation, not row crops. In these arid regions, just one millet plant per square meter is a common agronomic practice, but few yield models are designed to capture this kind of very low-density cultivation typical of marginal dryland ecosystems (Fig. 1).

Therefore, without high-quality information on field-level cultivation practices, precipitation, or impact of rainfall variability on yield, agricultural insurance programs will continue to suffer from significant basis risk, which is where climate index payout triggers are often unable to capture the actual loss experienced by the farmer. Although smallholder agricultural insurance (10) is one of many responses and adaptations to climate change, it typically suffers from low demand from farmers when offered on an open market (56). If Earth science data and agricultural models were better able to capture farmers’ losses due to climate variability, then agricultural insurance would have lower basis risk and potentially greater uptake and impact on socioeconomic risks of the community.

## Case Study #2: Choosing a Climate Indicator

When faced with an extreme climate event, decision-makers need to choose climate indicator(s) that detect the hazard(s) and capture the severity and extent of their impacts. The choice of climate indicator depends on the physical impacts of the hazard(s), the kind of decision that needs to be made, the role and level of expertise of the decision-maker, and the urgency with which they are required. For example, if the hazard is extremely high temperatures during a heat wave, temperature data from stations in urban areas with little vegetation and the potential for higher temperatures at night could be most relevant (57). However, it may be that humidity or soil moisture may be the most important parameter that will help a decision-maker determine what actions to take (58), for example, to warn of an increased probability of flooding when soils are saturated before a rain event. New York City’s Office of Emergency Management provides clear information notifications to residents about how they can stay cool during heat-related emergencies, and where cooling centers are located (59). In this case, the definition and quantitative triggers of heat emergencies are codified in legislation and accounted for in budgets, procedures, and plans of implementing agencies. However, other regions lack specific guidance, so health workers and government officials need to choose which indicator is most relevant.

For example, the 2003 drought and series of heatwave events in Europe were characterized by exceptional anomalies of temperature, rainfall, and vegetation. For example, the marked reduction of European crop yields during that year was attributed to the extreme heat and severe drought conditions. At the time of the event, governments were not prepared and had no procedures or protocols in place. Although in retrospect, the event was historic, with many multisectoral impacts:

- Temperature. The highest positive anomalies of average monthly air temperatures for the period of June-August 2003 exceed the long-term average (1961–1990 baseline/climatology) by up to 5 °C (60). The most extreme part of this heatwave event occurred in the first half of August. Through analysis of anomalies, Rebetez et al. (61) established that the 2003 drought and heatwave event in Europe was of exceptional severity and duration.
- Rainfall. Combined with a severe rainfall deficit in 2003, intensely drier-than-usual conditions with low relative humidity and high solar radiation developed over a prolonged period, exacerbating negative impacts on crop growth and yield outcomes (61). The impact of prolonged drier-than-usual conditions for the previous two years was compounded by high evaporation rates due to increased sunshine duration and elevated temperatures (62).
- Vegetation. Analysis of impacts on vegetation using anomalies of remote sensing data on vegetation indices (relative to a baseline of 1988–2002 average conditions) showed that in March 2003 the drought was already detectable in parts of western Europe, in April—in central and Eastern Europe, and in central France particularly from June onward. Recovery of vegetation was finally seen in early 2004.
- Soil moisture: The months leading up to the extreme summer event of 2003 were marked by a significant shortfall in rainfall, coinciding with an unusually early greening of vegetation and substantial positive radiative anomalies. This combination set off a complex feedback loop, resulting in an early and steep decline in soil moisture levels, far surpassing the multiyear average. Due to the severe depletion of soil moisture, latent cooling was greatly diminished, further exacerbating the surface temperature anomalies.

**Fig. 3.**
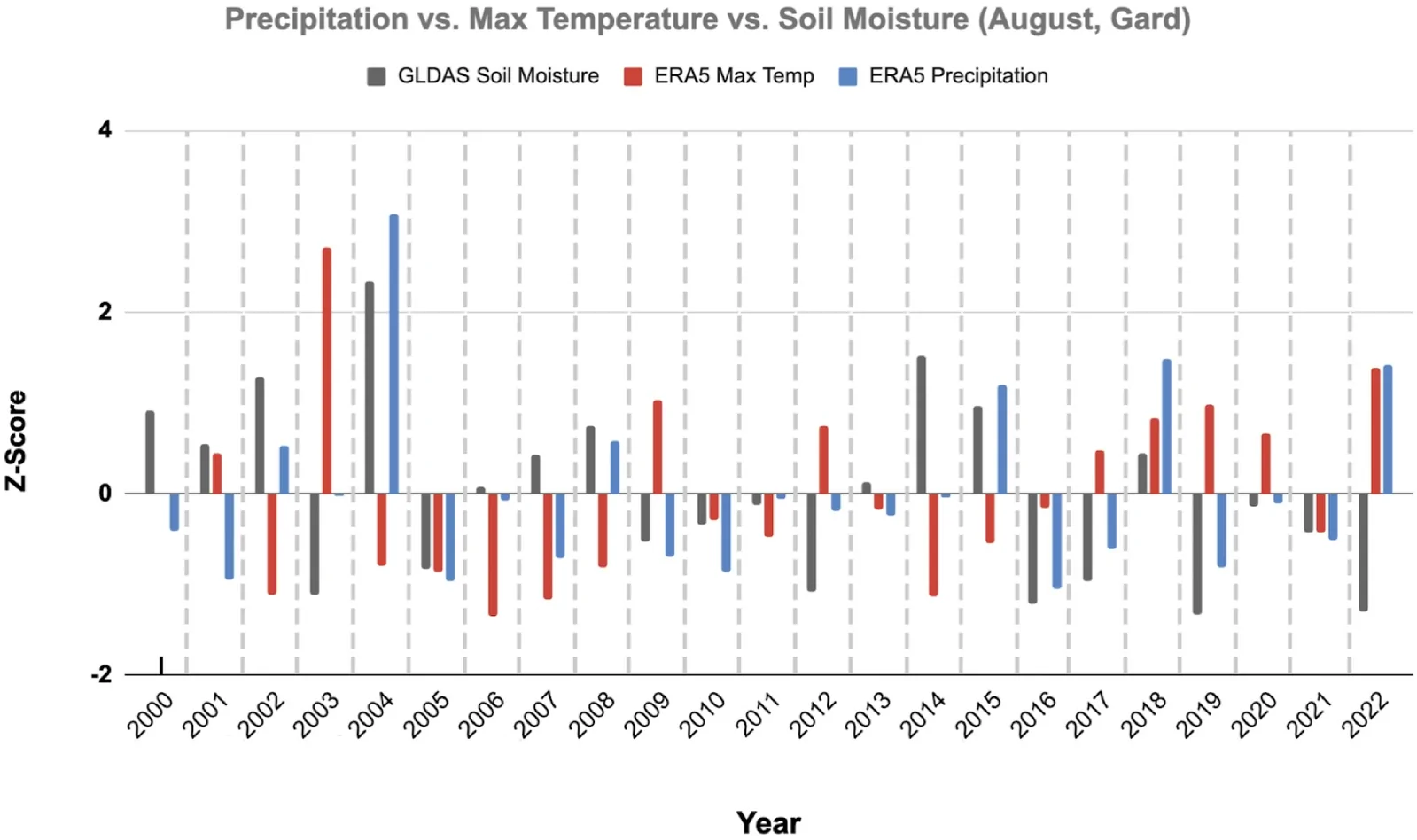
Soil moisture from the Global Land Data Assimilation System (GLDAS), maximum temperature, and precipitation from ERA5 (63) z-scores for the Gard Department (France) for August of each year from 2000 to 2022.

To capture all these aspects, we need measures of soil moisture, temperature, and precipitation together at the same location and time period. Fig. 3 shows the Z-score (which is the deviation from the mean adjusted for SD/variance) for datasets on temperature, precipitation, and soil moisture focused on capturing the impact of the heat wave on moisture deficit in one of the most affected regions, Gard in southern France, for August 2003. The figure illustrates that although different datasets can detect the anomaly, they document different severities and at different times. If one variable shows a severe anomaly during a certain period and the other one does not, this alone does not indicate which is right or wrong.

However, it means that further analysis and/or interpretation is needed. Using different sensors or models limits the likelihood that the same model error propagates into different variables. Fig. 3 might initially seem confusing to decision-makers because it does not show a strong negative rainfall anomaly next to the strong positive temperature anomaly in the same period. This is because the 2003 event in France was driven mostly by a heatwave, together with a slightly below-normal rainfall. The negative soil moisture anomaly during August 2003 confirms that the high atmospheric moisture demand (caused by extreme temperatures and related evapotranspiration) depleted water resources in the shallow soil layers.

In many cases, showing that different climate variables are “connected” requires an in-depth understanding of factors, such as time lags and feedback mechanisms. Local NWHS can provide this context to ensure appropriate decision-making during climate impact analysis. Depending on factors such as land surface type or antecedent climate conditions, the correlation coefficient between different climate variables tends to increase if the “right” time lag is considered. Transforming the raw data into Z-scores allows direct comparisons between variables across time and space (for example, temperature can be compared with precipitation in terms of magnitude of the anomaly) and limit the cognitive bias (e.g., temperature >30 °C is “critical”) due to the inherent standardization of the underlying data. They provide a way to contextualize anomalies to have spatial relevance and improve their interpretability by decision-makers.

## Case Study #3: Choosing a Climate Baseline

Understanding socioeconomic impacts of climate extremes requires both an articulation of the climate anomaly relative to baseline conditions to define the severity and spatial extent of impacts, and engagement with government agencies responsible for crafting a response. A baseline is the mean against which current periods are compared to identify when, how strong, and where extremes are occurring. Context is critical in determining and interpreting the magnitude of an anomaly, the baseline of interest, and how the anomaly is presented, to link it to socioeconomic impacts.

To understand potential impacts of a climate anomaly, local NWHS and other actors typically use ground-based observations from weather stations and ground radar, satellite data, and/or models, to derive climatological averages for regions of interest and over different periods of time. Determining whether a current or forecasted event is sufficiently anomalous to warrant a governmental response depends on previous experience with multisectoral impacts of hazards, deviations from averages, and spatial extent of impacts.

The drought and heatwave of 2003 in Europe posed a twofold challenge for agricultural production and public health and highlights the importance of baseline choice to define societally relevant anomalies.

For timely and accurate crop yield forecasting, monthly bulletins by the European Commission Monitoring Agricultural ResourceS (MARS) rely on anomalies of relevant agro-meteorological indicators calculated at different time steps (10-day, month, season, year, decade) against different baselines. This allows anomalies of short- (recent 5 y), medium- (recent 10 y), or long-term (30+ y) to be put into context, depending on the data source and specific objectives. For example, estimating the impact of a drought on crop yield should not include years so far in the past that the yield trends due to changes in agricultural technology (such as hybrids and fertilization intensity) could be larger than the impact of weather extremes (64). Consistent with this, the MARS agrometeorological analysis of 2003 resulted in yield forecasts that were up to 21% lower than the previous year and up to about 20% lower than the previous 5-y yields for all key crops. The substantial reduction of European yields was explained by the severe drought conditions and extreme heat stress (also on crops) in 2003 (65, 66).

On the other hand, the heatwave in France in 2003 resulted in over 15,000 deaths in the first fifteen days and numerous other negative impacts over the subsequent months (67). Although the high temperatures were forecasted, the French weather agency Météo-France did not anticipate the length or severity of the event, nor the impacts it would have on urban residents. The event led to the first national heatwave plan for France in May 2004 which set up a weather alert service, created a registry of people at risk, and articulated response guidelines for hospitals and voluntary aid workers if a heatwave was forecasted (68). This foundation has led to a much broader heat warning system that is jointly operated by the French public health agency Santé publique France, and the French weather agency Météo-France which is in place today.

The system defines heatwaves to be periods when the minimum and maximum temperatures averaged over 3 d exceed local thresholds (69). These maximums must exceed the 99.5th percentile of the 1981–2010 temperature averages. Upon declaring a heatwave, the government launched an ambitious communications plan that includes behavioral advice and short-term changes in lifestyle to respond to the warm conditions, including modifying working hours, canceling outdoor sportive activities, organizing freshwater distribution in public transportation, and opening public spaces during the night (69). Additional resources are diverted to hospitals, funds are spent on cooling centers, and health professionals are deployed to check on the vulnerable.

These responses, however, are expensive, and distributing these where they are most needed is essential to the success of an effective climate change response/adaptation effort. The spatial extent of the event is different with a different baseline, so setting a baseline with a quantitative basis allows decision-makers to distribute costly and limited resources to where they are most urgently needed since the anomaly map provides info on both magnitude of deviation from normal and spatial extent of impacts at different magnitudes.

To declare a heat event, selecting the time step and baseline period as a first step helps to determine whether the event reaches the threshold to declare a heat emergency. For meteorological data, the baseline temperature is typically computed by averaging 30 or more years of temperature data. A positive anomaly indicates the observed temperature during the period of interest was higher than the baseline (warmer-than-average conditions), while a negative anomaly indicates the observed temperature was lower than the baseline (cooler-than-average conditions).

**Fig. 4.**
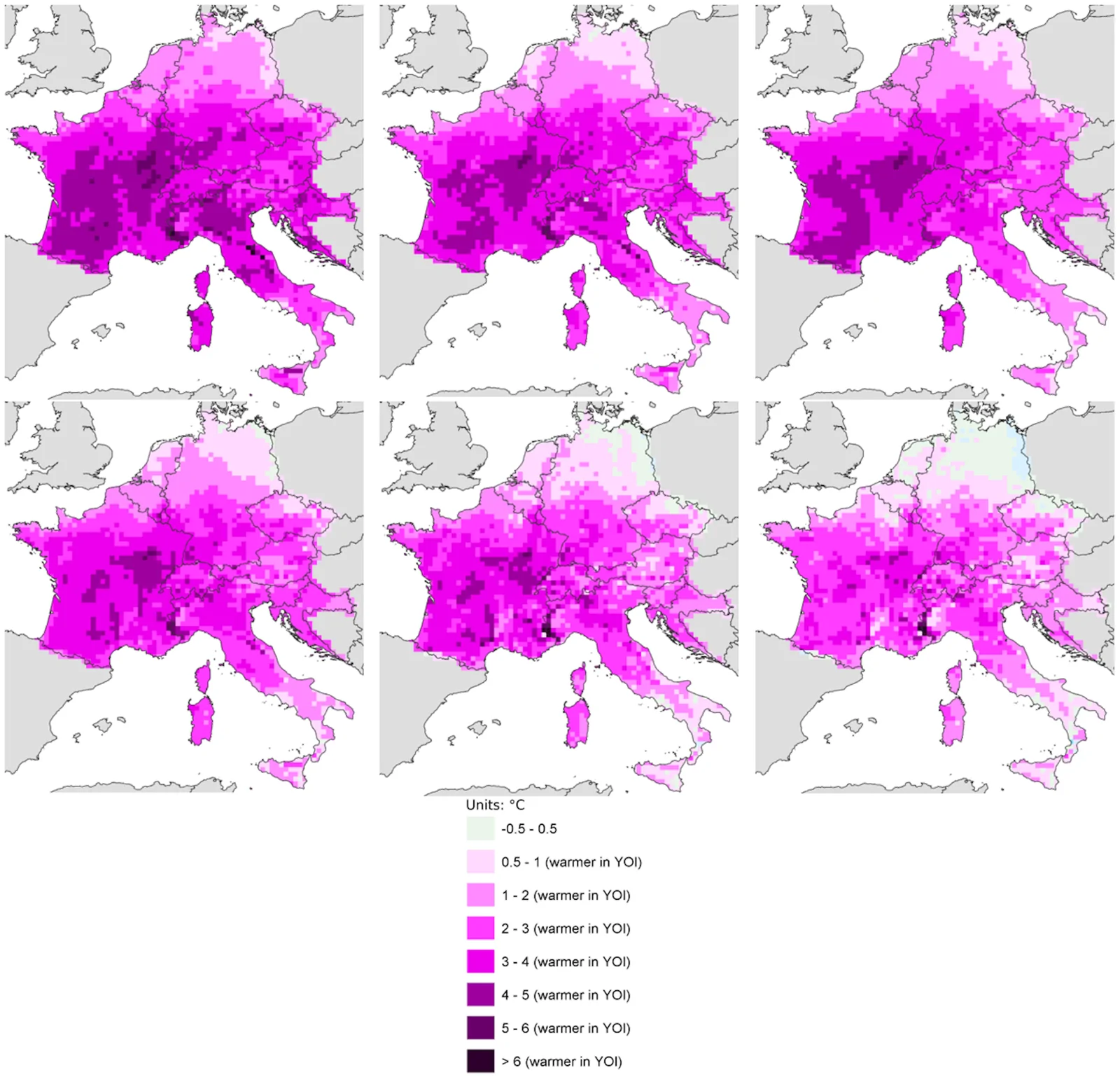
Average daily temperature anomalies for August 2003 [the Year of Interest (YOI)] relative to, *Left* to *Right*, the long-term, 10-y, and 5-y average, i.e., 1979–2002, 1993–2002, and 1998–2002 baseline (*Top* row) and relative to a 1991–2022, 2013–2022, and 2018–2022 baseline (also *Left* to *Right*, bottom row). Note: The reference period (baseline) reflects a warming trend.

From a hindsight perspective, we illustrate the importance of choosing a relevant baseline for defining an anomaly and determining the severity and spatial extent of impacts. In Fig. 4, we illustrate using the MARS AGRI4CAST meteorological observations database to show how the length of the baseline period affects how strongly the 2003 heatwave appears in the data. This has a twofold meaning—on one hand, the magnitude of the anomaly indicates the severity of the event, while on the other hand, the extent of the impacts can be defined according to the spatial extent of the anomaly at different severity levels, thus helping analysts and decision-makers to understand impacts in terms of croplands and populations affected. The 2003 event was notable at the time, but when it is compared to a 5-y mean, most decision-makers would likely not interpret it as extreme, because the 2018–2022 period was hot and dry. If a 30-y reference period is used, with the last 5 y being above normal, the 2003 event would be interpreted as extreme. In the world of trends, one needs to be aware of the impact of the current year on the system. With limited resources, analysis according to different baseline periods such as these in Fig. 4 helps decision-makers to allocate resources to where they are most urgently needed based on the magnitude and extent of the temperature anomaly.

With the coupled hazards of heatwave and drought in 2003 in Europe as an example, we show how different baseline periods could be useful depending on the decision-making objective. Choosing a baseline that is relevant to the hazard(s) helps decision-makers to identify areas most severely affected and thus, to optimally distribute (often limited) response resources.

## Discussion and Conclusions

As the climate warms, the impacts on human health (70), natural ecosystems (71), and agricultural production (72) are likely to intensify. However, these impacts will not be uniform in time or across regions and some places will be far more affected than others. Understanding how to capture and analyze the impact of rising temperatures and related climatic feedback loops, such as increasing evaporative stress or soil moisture depletion, is essential for social scientists, economists, agronomists, and others without specific expertise on selecting or analyzing climate data.

Here, we present three different cases of how data quality, variable selection, and different baseline periods could affect the response to and perception of the event. Policymakers can be influenced by maps and analyses that show more or less severe impacts on a community, or be more prone to responding if some areas are affected instead of others (73). How data are presented and interpreted drives the urgency with which policymakers respond to climate hazards. Although funds have been pledged to compensate countries and communities who experience loss and damage due to extreme weather, the amount pledged is far less than what is needed (74). Anticipating climate impacts can reduce the loss of life (75), but without concerted transformation of adaptation and mitigation efforts, these losses are likely to accelerate.

Ciais et al. (66) showed through continent-scale analysis of carbon-climate feedback and vegetation response to heatwave and drought conditions using historical crop yields, that rainfall deficit and extreme heat explain the unprecedented gross primary productivity reduction in Europe during the summer of 2003. The work of local NWHS is critical for determining how extreme the event is and for maintaining weather observations necessary to create a clear baseline. Because the NWHS agencies can act within the government and motivate appropriate health, safety, and private sector responses, their role is essential.

The United Nations has launched a new initiative called “Early Warnings for All,” where everyone on Earth is provided access to life-saving early warning systems by the end of 2027. The Global Commission on Adaptation estimated in 2019 that a 24-h notice of an impending disaster can reduce damage by 30 percent. Investing $800 million in such systems in developing countries would prevent losses of $3 to $16 billion annually (76). In such a global early warning system, we will need to know what is extreme for each location.

Building an “early warning system for all” requires being very specific about the hazards that may be experienced in any one place. Is it a short-term hazard like a flood, or a longer-term hazard like a drought that may take a year or more to be replaced with more normal climate conditions? In parallel, the characteristics of different hazard types are changing, making events like flash droughts much more likely under the impact of climate change (77). If the hazard is something that kills you directly and immediately, it is easier to justify intervention because the forecast leads directly to the action you need to take. The shifting underlying probabilities due to climate change complicate the understanding and communication of the likelihood of extreme events. Improved data quality, climate indicators, and baseline selection will improve communication of how climate can lead to high socioeconomic losses/costs that early investments into adaptation and infrastructure can mitigate.

## Data Availability

Previously published data were used for this work (63).
